# Advances and Challenges in Biopolymer-Based Films

**DOI:** 10.3390/polym14183920

**Published:** 2022-09-19

**Authors:** Swarup Roy, Jong-Whan Rhim

**Affiliations:** 1 School of Bioengineering and Food Technology, Shoolini University, Solan, Himachal Pradesh 173229, India; 2 Department of Food and Nutrition, BioNanocomposite Research Institute, Kyung Hee University, 26 Kyungheedae-ro, Dongdaemun-gu, Seoul 02447, Korea

Today, biobased polymers derived from sustainable and renewable natural sources are of great interest as an alternative to control the severe damage already caused by petro-chemical-based polymers. The extensive use of non-biodegradable plastics in the packaging sector produces an enormous amount of waste, ultimately ending up in landfills and the ocean. The scenario of packaging pollution has become more severe owing to the COVID-19 pandemic, and recently, many countries have to ban the use of single-use plastics, since worldwide yearly manufacturing of plastic materials is ~400 million tonnes; interestingly, about 40% of these materials are utilized for single-use packaging materials [1]. Thus, the present scenario urgently demands the replacement of synthetic plastics with biobased alternatives. The importance of biopolymers in packaging must be considered to provide a better and more sustainable future. Biopolymers are not a new concept and have been used since ancient times, but research on using biopolymers as a replacement for packaging materials began in the early 2000s. The use of biopolymers in developing packaging materials is a promising field of research as it comprehensively reduces plastic waste and decreases greenhouse gas emissions [2,3,4]. Varieties of biopolymers originating from renewable products and food waste, such as polysaccharides (cellulose, chitosan, pectin, carrageenan, agar, etc.), proteins (gelatin, soy protein isolate, zein, etc.), or their blends (gelatin/agar, chitosan/pullulan, pectin/agar, gelatin/zein, etc.), have been used in this regard for film production [5,6,7,8]. Biopolymers have great potential to replace conventional plastics due to their non-toxicity, biocompatibility, and fast degradability.

Moreover, biopolymers can make a good film with excellent physical properties. Furthermore, biobased polymers are good sources of the carriers of bioactive ingredients that can impart functionality to the packaging material to improve the food shelf-life of packed food [9,10]. Biopolymer-based film has been extensively used in fabricating various active and smart packaging films and coatings [11,12]. Current reports suggest that biobased-blend polymer-based packaging film showed comparable physical properties to convenient polymers-based film. Moreover, introducing active and intelligent packaging makes biobased polymers more popular in the packaging sector [13,14,15]. Even though the use of biopolymers is advantageous in many respects, especially to address plastic waste and food safety concerns, there are still many limitations compared to its counterpart, which need to be resolved to meet the requirement of synthetic plastics [16,17]. Synthetic plastics are easy to handle, cost-effective, highly flexible, and water-insoluble, which make them convenient for making a suitable product used in the packaging regime.

On the other hand, biobased polymers are costly and generally hydrophilic [18,19]. Therefore, improving hydrophobicity, cost minimization, and scale-up production of packaging film using biobased polymers could solve the drawbacks of biopolymers. As eco-friendly packaging materials, there are plenty of opportunities for biobased polymers in the food sector. The worldwide market price of biopolymers is increasing at a rate of ~6–7% [20]. Nevertheless, more research is still required to address the challenges related to biopolymers for their practicability as a potential material for food packaging films. 
